# Supplementary material for: Power generation from the interaction of a liquid droplet and a liquid membrane
Source: Nat Commun. 2019 May 22;10:2264. doi: 10.1038/s41467-019-10232-x (PMC6531479; doi:10.1038/s41467-019-10232-x)
Supplement: Supplementary file 1 — Supplementary Information [file 41467_2019_10232_MOESM1_ESM.pdf]

Supplementary Information

Power generation from the interaction of a liquid droplet and a liquid membrane

Nie et al.

Supplementary Figures

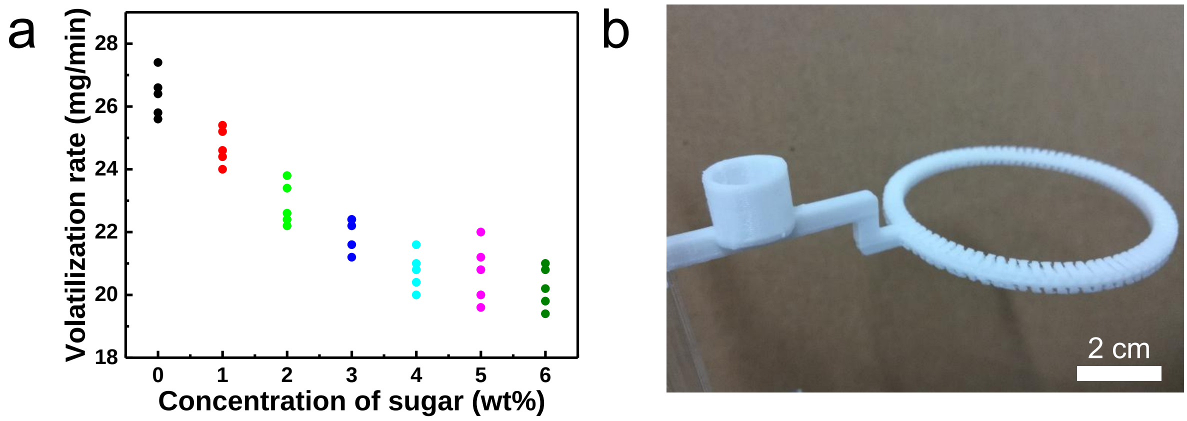

Supplementary Figure 1. a The evaporation rate of the liquid membrane at different sugar concentrations. The diameter of the tested liquid membrane is 60 mm, and the volatilization rate was tested for 5 times. **b** The structure of the reservoir is designed to extend the lifetime of the liquid membrane.

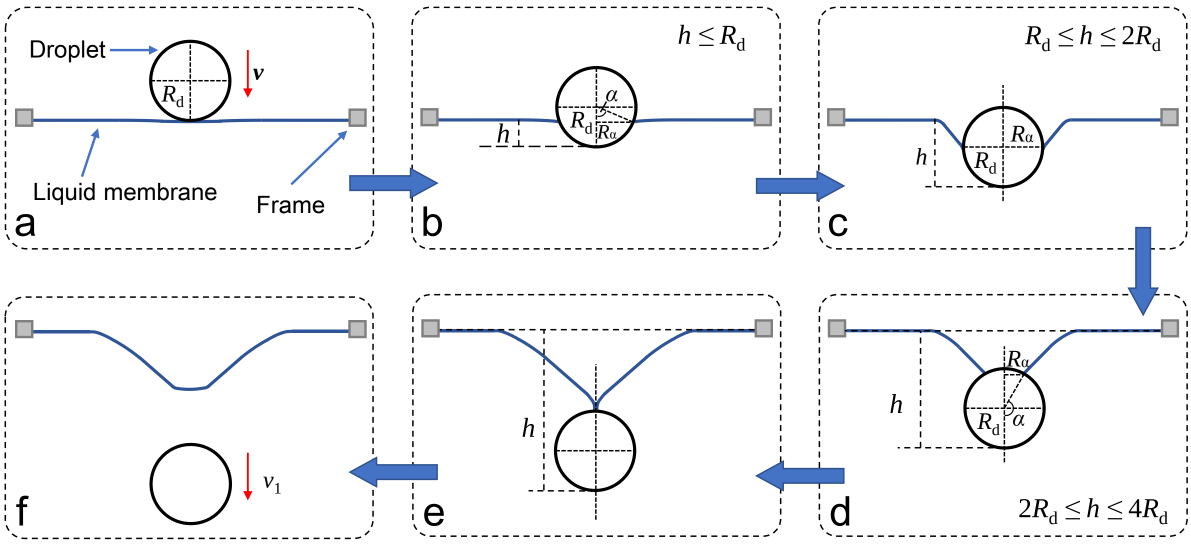

Supplementary Figure 2. Schematic diagram of a droplet passing through a liquid membrane.

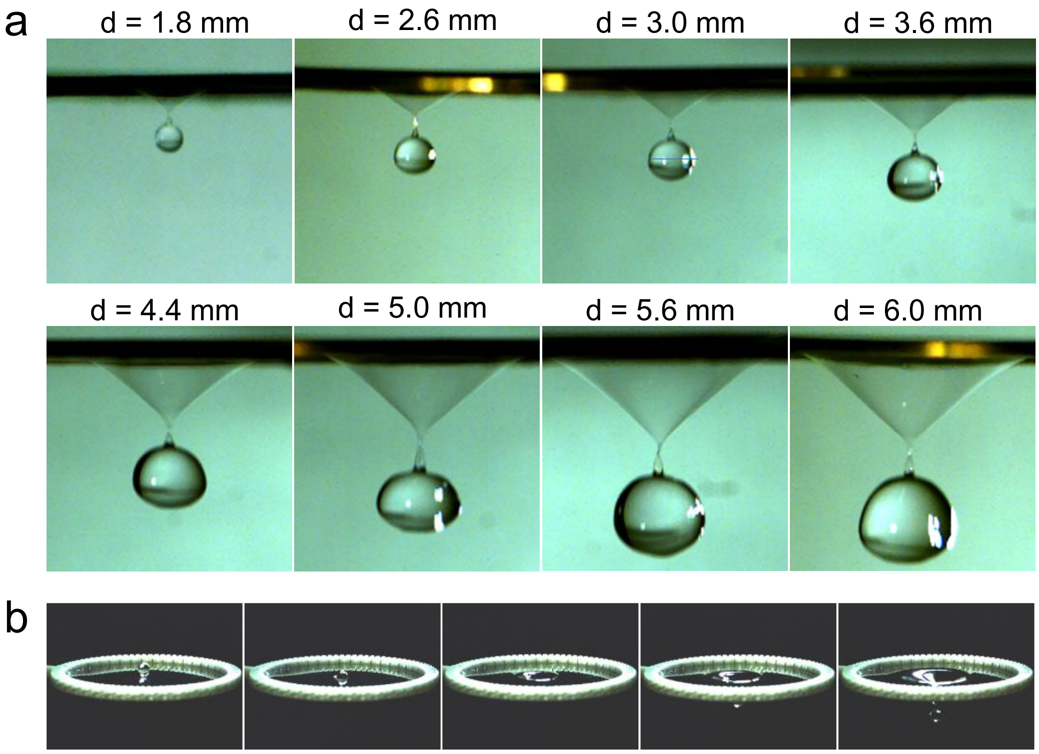

Supplementary Figure 3. a Photographs of different sizes of water droplets passing through the liquid membrane. **b** Images of a water droplet passes through the liquid membrane. The droplets fell from a height of 2 m.

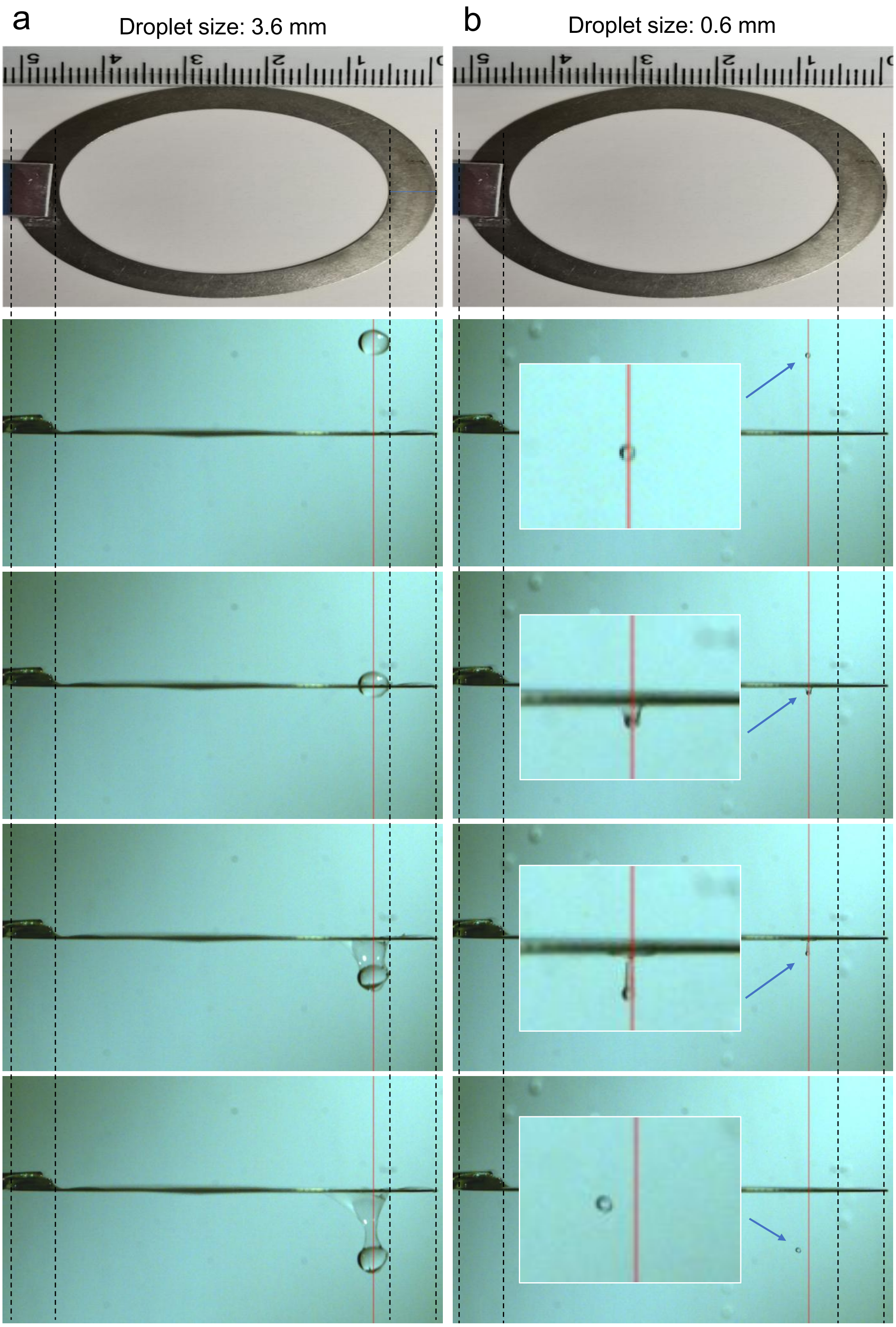

Supplementary Figure 4. Motion behavior of droplets passing through the edge region of membrane with the size of 3.6 mm (**a**) and 0.6 mm (**b**).

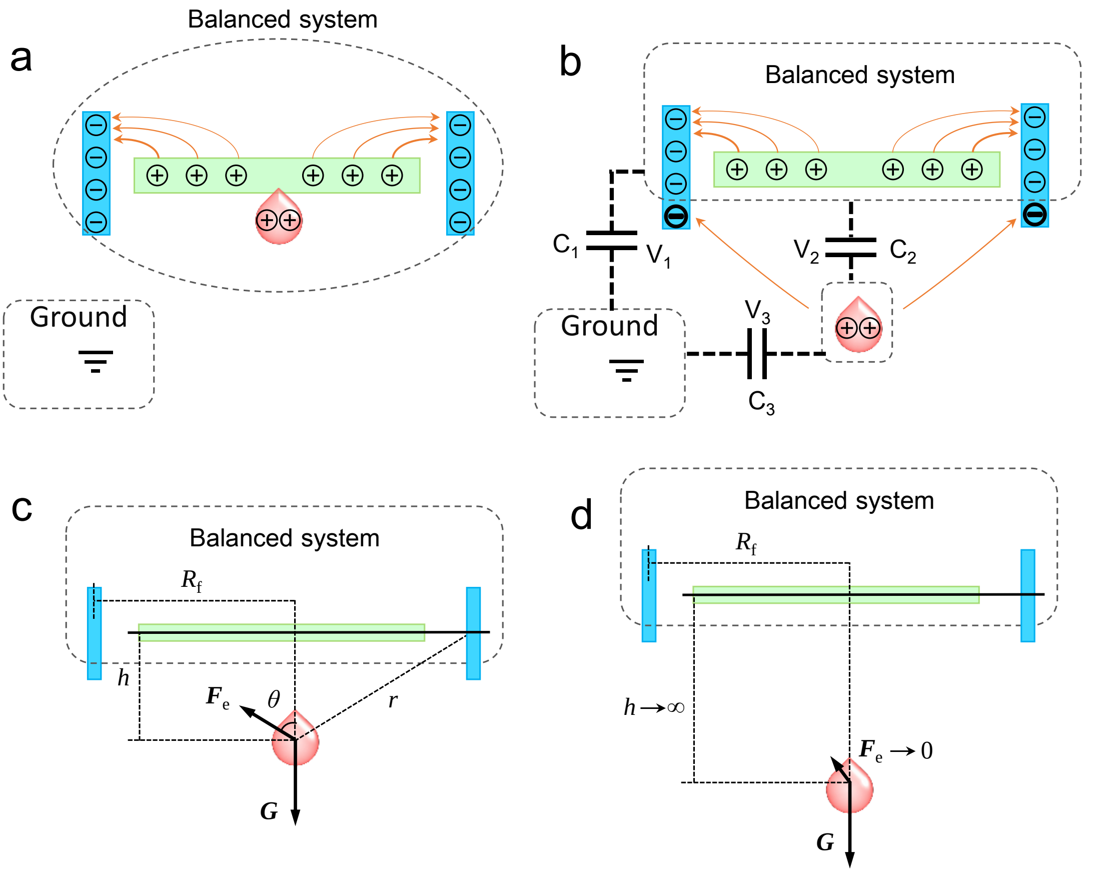

Supplementary Figure 5. The kinetic energy of droplet is converted into electrical energy. a Schematic diagram of electric field distribution with water droplet at different positions. **b** Circuit model with three capacitances for the liquid membrane-based nanogenerator at open-circuit condition. **c,d** The force analysis of the water droplet after passing through the liquid membrane.

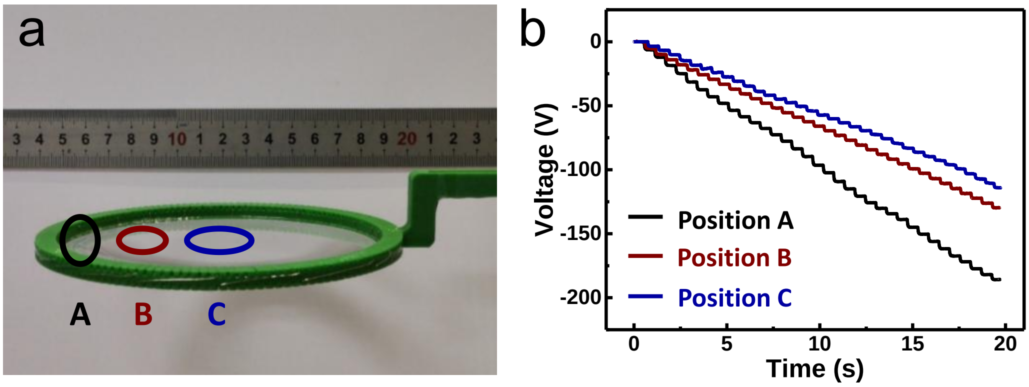

Supplementary Figure 6. **A larger frame is prepared for studying the different output at different falling position of the membrane. a** A larger membrane for checking the charge transfer and three different positions on the liquid membrane for droplets to pass through. **b** V_{OC} of droplets passing through these positions.

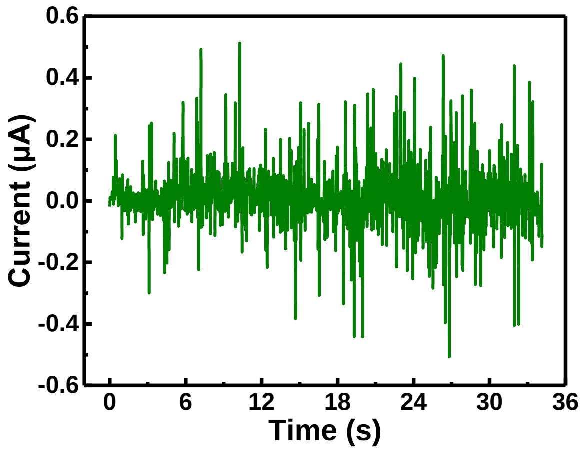

Supplementary Figure 7. I_{SC} of the real raindrops passing through the grounded liquid membrane.

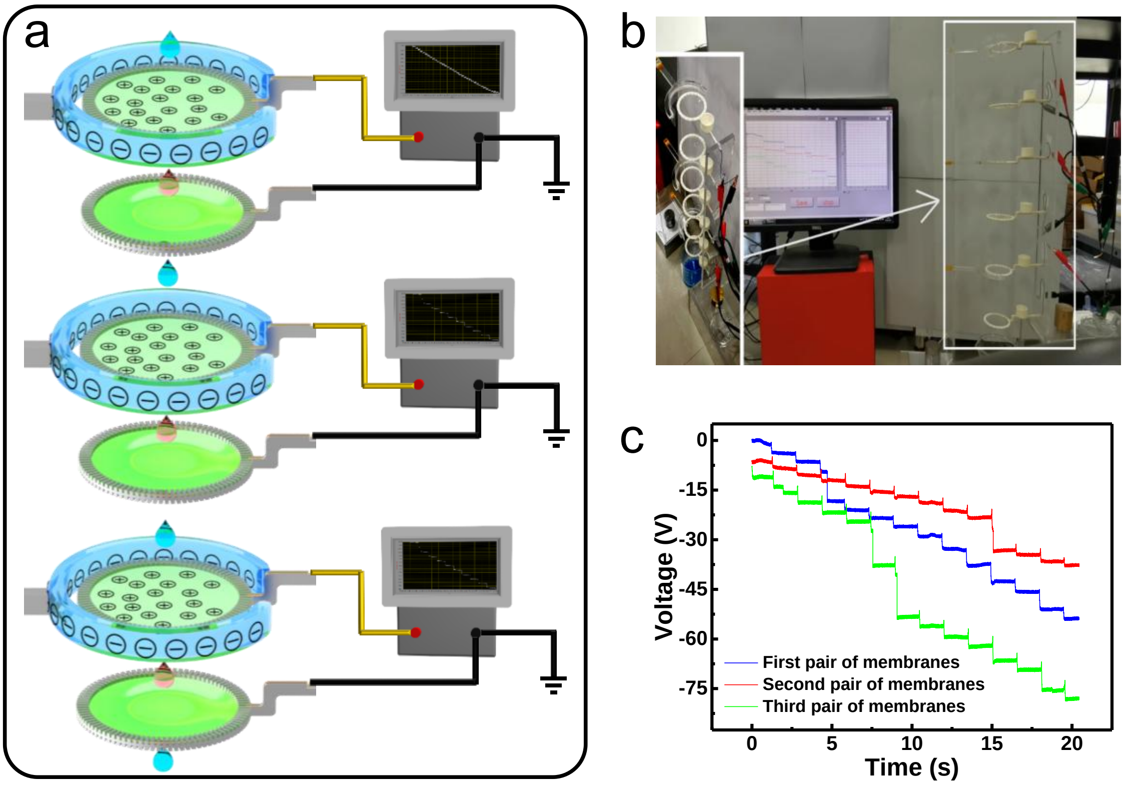

Supplementary Figure 8. A staking TENG system with six liquid membranes to harvest energy from falling droplets. a Schematic diagram of the staking TENG system with three electrometers monitoring the output signal. **b** Photograph of the test system. Insert is the close-up of the multiple pairs of membranes. **c** Output voltage of this staking TENG system. It is necessary to note that the droplet can carry a certain amount of charges after passing through the first pre-charged membrane. If these residual charges are not fully removed by the grounded membrane, they can disturb the energy generation of the other two pre-charged membranes and the electrometers may observe disordered signals. Hence, in our demonstration, the grounded membranes (the second, the fourth and the sixth) in this stacking system are connected to the grounding end of the electrometers. Meanwhile, the impact process of droplets can also take away some solution from the liquid membrane, which leads to the increase of droplet volume. Hence, we can observe some sudden increase of voltage amplitude during the measurements, as shown in Supplementary Figure 8c.

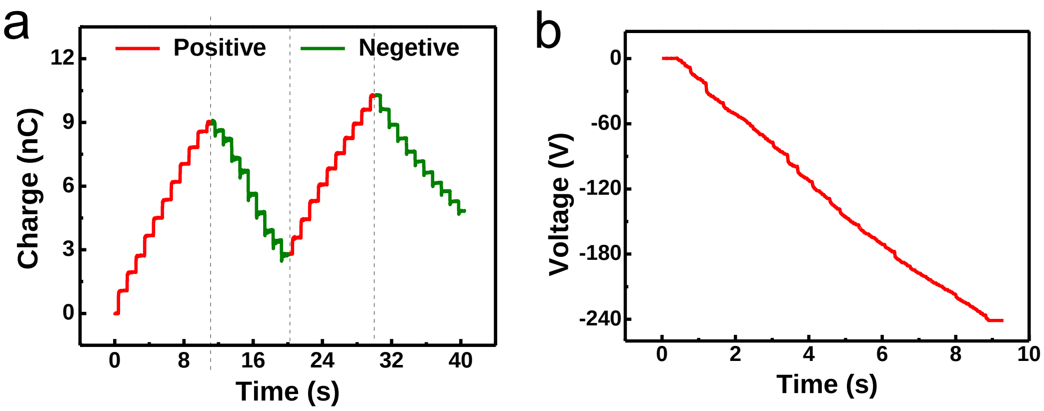

Supplementary Figure 9. a Transferred charges of water droplets passing through different locations of the polarized liquid membrane. **b** V_{OC} of the multi-tube droplets passing through a grounded liquid membrane in polarized mode.

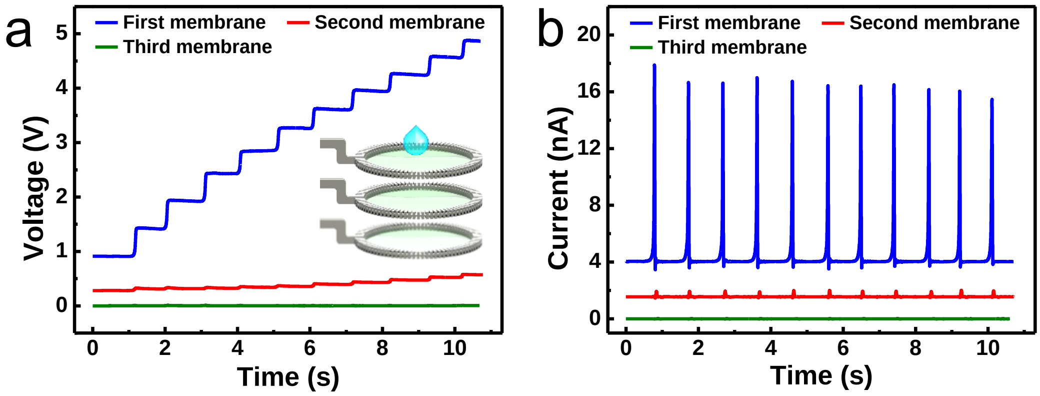

Supplementary Figure 10. **a,b** Open-circuit voltage (**a**) and short-circuit current (**b**) of droplets passing through three liquid membranes. Insert of (**a**) is the Schematic diagram of a droplet passing through three liquid membranes.

Supplementary Tables

Supplementary Table 1. Lifetime of the liquid membrane with different surfactants and different concentrations of sugar. The average lifetime (with standard deviations) were calculated from 3 independent measurements. (Test conditions: temperature 25 ± 0.5 ℃, humidity 30 ± 2 %, five drops of water per second through the liquid membrane)

**Concentration of sugar (wt%)**	**Lifetime of the liquid membrane (s)**		
	2.1 wt% AES + 0.5 wt% PVA	0.3 wt% SDS + 0.5 wt% PVA	6 wt% Walch + 0.5 wt% PVA
0	72 ± 11	65 ± 8	179 ± 21
1	94 ± 18	105 ± 16	173 ± 27
2	90 ± 13	170 ± 24	186 ± 30
3	82 ± 15	216 ± 19	189 ± 29
4	84 ± 20	257 ± 18	195 ± 27
5	86 ± 16	273 ± 28	179 ± 35
6	78 ± 18	281 ± 24	195 ± 32
8	74 ± 21	275 ± 30	169 ± 20
10	68 ± 18	265 ± 31	196 ± 24
15	60 ± 19	251 ± 27	195 ± 32
20	58 ± 14	233 ± 18	177 ± 28

Supplementary Table 2. Lifetime of the liquid membrane with different surfactants and different concentrations of PVA. The average lifetime (with standard deviations) were calculated from 3 independent measurements. (Test conditions: temperature 25 ± 0.5 ℃, humidity 30 ± 2 %, five drops of water per second through the liquid membrane)

**Concentration of PVA (wt%)**	**Lifetime of liquid membrane (s)**		
	2.1 wt% AES + 3 wt% sugar	0.3 wt% SDS + 5 wt% sugar	6 wt% Walch + 4 wt% sugar
0	56 ± 13	96 ± 9	77 ± 12
0.1	68 ± 18	176 ± 18	107 ± 18
0.2	74 ± 24	259 ± 25	159 ± 25
0.3	72 ± 25	291 ± 28	151 ± 26
0.4	76 ± 19	308 ± 34	182 ± 28
0.5	84 ± 16	294 ± 36	186 ± 20
0.6	82 ± 21	296 ± 33	192 ± 35
0.8	90 ± 28	258 ± 27	229 ± 34
1.0	80 ± 23	220 ± 31	195 ± 27
1.5	96 ± 26	236 ± 28	243 ± 37
2.0	82 ± 16	231 ± 33	241 ± 41

Supplementary Table 3. The viscosity and density of membrane solution with different concentrations of PVA. (Test environment: temperature 25.0 ± 0.3 ℃)

Concentration of PVA (wt%)	Viscosity (cP)	Density (kg/m^3)
0	1.079	1020.1 ± 1.5
0.1	1.276	1018.3 ± 0.8
0.2	1.326	1017.3 ± 1.2
0.3	1.356	1017.6 ± 0.6
0.4	1.417	1018.5 ± 2.2
0.5	1.478	1018.7 ± 1.8
0.6	1.554	1018.3 ± 1.4

Supplementary Table 4. Physical parameters of the optimized membrane solution (0.3 wt% SDS, 5 wt% sugar and 0.4 wt% PVA). Test environment: Temperature 25 ± 0.3 ℃, humidity 30 ±2 %, atmospheric pressure 101.2 kPa.

**Physical parameters**	**Viscosity (cP)**
Density (kg/m3)	1018.5 ± 2.2
Viscosity (cP)	1.417
Vapor pressure (kPa)	0.363
Conductivity (μS)	559 ± 6
Surface Tension (mN/m)	33.57 ± 0.04

Supplementary Table 5. The change in velocity of rain droplets of different sizes as they pass through the liquid membrane. (Calculation results)

**Diameter of drops (mm)**	**Through the liquid film**	**Decrease of velocity**
0.1	×	100%
0.2	×	100%
0.4	√	12.96%
0.6	√	5.18%
0.8	√	2.82%
1.0	√	1.61%
1.6	√	0.61%
2.0	√	0.32%
2.6	√	0.22%
3.0	√	0.16%
4.0	√	0.069%
5.0	√	0.065%
5.8	√	0.052%

Supplementary Notes

Supplementary Note 1: Analysis of velocity of raindrops passing through the liquid membrane.

When a droplet passes through a liquid membrane, the surface tension from the liquid membrane will reduce the velocity of the droplet. Meanwhile, for the droplets with high velocity, the effect of air resistance on velocity is also not negligible. As discussed in the main text, the water droplet is subjected to gravity (G), surface tension (F_s) and air resistance (F_a). The force analysis of water droplet can be expressed by the following equation:

$$G+F_{\mathrm{s}}+F_{\mathrm{a}}=ma \tag{1}$$

where m is the mass of the droplet, and a is the acceleration. G can be expressed as:

$$G=mg \tag{2}$$

where g is the gravity acceleration. F_s is expressed as:

$$F_{\mathrm{s}}=-4\pi R_{\alpha}\gamma\sin\alpha=-4\pi\gamma\frac{R_{\alpha}^{2}}{R_{\mathrm{d}}} \tag{3}$$

where R_α is the radius of the circle where the water droplets contact the liquid membrane (Figure S 2b), R_d is the radius of the droplet, and γ is the surface tension coefficient. F_a is expressed as:

$$F_{\mathrm{a}}=-\frac{1}{2}cv^{2}\cdot\pi R_{\mathrm{d}}^{2} \tag{4}$$

where c is the air resistance coefficient, v is the velocity of the droplet. Thus, substituting Equations 2−4 into Equation 1 can obtain the following equation:

$$mg-4\pi\gamma\frac{R_{\alpha}^{2}}{R_{\mathrm{d}}}-\frac{1}{2}cv^{2}\cdot\pi R_{\mathrm{d}}^{2}=ma \tag{5}$$

To determine the effect of the liquid membrane on the falling speed of droplet, v and a are replaced with a differential of the drop height (h) and drop time (t), as shown in the following equation:

$$mg-4\pi\gamma\frac{R_{\alpha}^{2}}{R_{\mathrm{d}}}-\frac{1}{2}c(\frac{\mathrm{d}h}{\mathrm{d}t})^{2}\cdot\pi R_{\mathrm{d}}^{2}=m\frac{\mathrm{d}^{2}h}{\mathrm{d}t^{2}} \tag{6}$$

where R_α is a function of h. When the droplet is just in contact with the liquid membrane, h = 0 m, t = 0 s, as shown in Supplementary Figure 2a. When water droplet is in contact with the liquid film, R_α is approximated as a function of h and R_d, as shown in the following equation:

$$R_{\alpha}(h)=\begin{cases}\sqrt{R_{\mathrm{d}}^{2}-(R_{\mathrm{d}}-h)^{2}}, & h\le R_{\mathrm{d}}\\ R_{\mathrm{d}}, & R_{\mathrm{d}}<h\le 2R_{\mathrm{d}}\\ \dfrac{3R_{\mathrm{d}}-h}{2}+\dfrac{1}{2}\sqrt{2R_{\mathrm{d}}^{2}-(3R_{\mathrm{d}}-h)^{2}}, & 2R_{\mathrm{d}}<h\le 4R_{\mathrm{d}}\end{cases} \tag{7}$$

Here, a few approximate relationships were applied based on the video of the water droplets passing through the liquid membrane. When h is less than R_d, the liquid membrane that does not come into contact with the water droplet does not move (Supplementary Figure 2b). When h is greater than R_d and less than

$2R_d$, R_α is assumed to be equal to R_d and remains the same value (Supplementary Figure 2c). When h is greater than $2R_d$ and less than $4R_d$, we assume that the liquid membrane is stretched at an angle of 45 degrees from the horizontal and the angle remains the same value (Supplementary Figure 2d). Then, substituting Equations 7 into Equation 6, the relationship between h and t can be expressed by the following equations:

$$mg - 4\pi\gamma \frac{R_d^{\ 2} - (R_d - h)^2}{R_d} - \frac{1}{2} c (\frac{dh}{dt})^2 \cdot \pi R_d^{\ 2} = m \frac{d^2 h}{dt^2} \tag{8}$$

$$mg - 4\pi\gamma R_d - \frac{1}{2} c (\frac{dh}{dt})^2 \cdot \pi R_d^{\ 2} = m \frac{d^2 h}{dt^2} \tag{9}$$

$$mg - \frac{4\pi\gamma}{R_d} [\frac{3R_d - h}{2} + \frac{1}{2}\sqrt{2R_d^{\ 2} - (3R_d - h)^2}\,]^2 - \frac{1}{2} c (\frac{dh}{dt})^2 \cdot \pi R_d^{\ 2} = m \frac{d^2 h}{dt^2} \tag{10}$$

It is experimentally measured that γ is 35mN/m, the different diameters of the water droplets correspond to different air resistance coefficients[1]. The curve of h-t is obtained by the numerical method through the MATLAB, and the relationship between v and t is obtained by deriving the derivative of h. When the droplet is separated from the liquid membrane (h=4 R_d), the change in velocity of droplets with different sizes can be calculated, as shown in Supplementary Table 4.

Supplementary Note 2: Theoretical calculation of open-circuit voltage (V_{OC}).

The charging and discharging of this liquid membrane is quite similar to the operation principle of a single electrode TENG[2]. In the process of water droplet away from the liquid membrane, the liquid membrane and the FEP film are regarded as a balanced system. As shown in Supplementary Figure 5b, the total charges on droplet, balanced system and ground are q, $-q$, and 0, respectively. The entire system of this membrane-based nanogenerator has been simplified as a physical model with three capacitors. At open-circuit condition, based on the basic characteristics of capacitance and charge conservation at each position, the following equation can descript the relationship between V_i (i = 1, 2, 3) and corresponding C_i,

$$V_3 = V_1 + V_2 \tag{11}$$

$$V_3 C_3 + V_2 C_2 = q \tag{12}$$

$$-V_1 C_1 + V_2 C_2 = -q \tag{13}$$

Combining Equations 11-13, the V_{OC} can be obtained as the following equation:

$$V_{OC} = V_1 = \frac{qC_3}{C_1 C_2 + C_2 C_3 + C_3 C_1} \tag{14}$$

When the drop height (h) of water droplet approaches to infinity, the value of C_2 and C_3 are approaching 0, and the ratio of C_2/C_3 approaches to 1. Therefore, the maximum V_{OC} can be obtained at infinite h, as shown in the following result:

$$V_{OC} \approx \frac{q}{2C_1} \tag{15}$$

Supplementary Note 3: Analysis of the electrostatic force on water droplets.

After separation from the liquid membrane, the charged water droplet is treated as a point charge with a charge of q. During the process of moving away from the liquid membrane, the droplet receives the combined electrostatic force from the liquid membrane and the FEP film. The effective charges (Q) on the balanced system to analyze the resultant electrostatic force is a function of h. Thus, the electrostatic force (F_e) applied on the falling droplet (Supplementary Figure 6c) is expressed as the following equation:

$$F_{\mathrm{e}} = \frac{qQ(h)}{4\pi\varepsilon_0 r^2}\cos\theta \qquad (16)$$

Where ε_0 is the permittivity of vacuum, r is the distance between the droplet and the edge of FEP film, θ is the angle between the direction of the drop and r (Supplementary Figure 6c). The r and cosθ can be given by:

$$r = \sqrt{h^2 + R_{\mathrm{f}}{}^2} \qquad (17)$$

$$\cos\theta = \frac{h}{r} = \frac{h}{\sqrt{h^2 + R_{\mathrm{f}}{}^2}} \qquad (18)$$

where R_f is the radius of the FEP film. Then substituting Equations 17 and 18 into Equation 16 can obtain the electrostatic force of droplet, as shown inn following equation:

$$F_{\mathrm{e}} = \frac{qQ(h)}{4\pi\varepsilon_0}\frac{h}{(h^2 + R_{\mathrm{f}}{}^2)^{\frac{3}{2}}} \qquad (19)$$

Supplementary References

1. Gunn, R. & Kinzer, G. D. The Terminal Velocity of Fall for Water Droplets in Stagnant Air. *J. Meteorol.* **6**, 243-248 (1949).
2. Niu, S., et al. Theoretical Investigation and Structural Optimization of Single-Electrode Triboelectric Nanogenerators. *Adv. Funct. Mater.* **24**, 3332-3340 (2014).
