# Supplementary material for: Power generation from the interaction of a liquid droplet and a liquid membrane
Source: Nat Commun. 2019 May 22;10:2264. doi: 10.1038/s41467-019-10232-x (PMC6531479; doi:10.1038/s41467-019-10232-x)
Supplement: Supplementary file 7 — Description of Additional Supplementary Files [file 41467_2019_10232_MOESM7_ESM.pdf]

Description of Additional Supplementary Files

File Name: Supplementary Movie 1
Description: Longevity of liquid membrane under a continuous water flow

File Name: Supplementary Movie 2
Description: Add 1 mL of solution every 5 min for long-term use of liquid membrane

File Name: Supplementary Movie 3
Description: Water droplets pass through the polarized liquid membrane to generate electrical energy continuously

File Name: Supplementary Movie 4
Description: Liquid membrane as an electrostatic filter for various objects
